# Botulinum Toxin in Parkinson’s Disease Tremor: A Critical Evaluation of the Evidence and Clinical Practice

**DOI:** 10.3390/toxins18070280

**Published:** 2026-06-25

**Authors:** Shivam Om Mittal, Wolfgang H. Jost

**Affiliations:** 1 Movement Disorders Center, Cleveland Clinic Abu Dhabi, Abu Dhabi 112412, United Arab Emirates; neurology.mittal@gmail.com; 2 Parkinson-Klinik Ortenau, 77709 Wolfach, Germany

**Keywords:** botulinum toxin, Parkinson’s disease, tremor, essential tremor, EMG-guided injection, incobotulinumtoxinA, ultrasound guidance, individualized technique, critical appraisal

## Abstract

Approximately 30% of patients with tremor-dominant Parkinson’s disease (PD) have rest tremor that persists despite optimal dopaminergic therapy. When deep brain stimulation and focused ultrasound are unavailable or declined, the therapeutic options narrow. Botulinum toxin (BoNT) offers a targeted, titratable, reversible approach, but whether a peripheral neuromuscular blocking agent makes sense for a centrally generated tremor is a legitimate question that deserves a direct answer. This narrative critical review appraises what is currently known across PD and non-PD tremor conditions, defines the technical requirements for safe and effective injection, and provides a practical framework for patient selection and clinical management. The PD-specific literature rests on a single positive double-blind randomized controlled trial of 30 patients; all remaining data are open-label or extrapolated from other tremor conditions, and this narrative synthesis combines heterogeneous conditions, outcome scales, and toxin protocols. A recurring technical observation is that, in the available trials, individualized, EMG-guided injection has been associated with substantially lower rates of hand weakness than fixed-dose injection (reported reductions from roughly 30–70% to below 15%) while maintaining tremor reduction, although the degree of benefit and weakness risk vary with the tremor syndrome, injected muscles, baseline impairment, dose, and guidance method. The careful patient selection this approach requires helps the individual clinician and patient achieve tremor relief, but it departs from the unselected real-world PD population and introduces selection bias that makes a large, statistically representative cohort difficult to assemble. In well-selected patients at centers with the appropriate expertise, BoNT may be a clinically useful option, but routine adoption is not yet supported.

## 1. Introduction

Rest tremor in PD is often the most treatment-resistant motor feature. Levodopa and dopamine agonists are the first-line, evidence-based therapy and control it adequately in the majority of patients, but a substantial proportion continue to have functionally significant rest tremor despite optimized dopaminergic therapy [1,2]. For those patients, deep brain stimulation (DBS) targeting the ventral intermediate thalamic nucleus or the subthalamic nucleus and MRI-guided focused ultrasound (FUS) thalamotomy of the ventral intermediate nucleus are both highly effective, but they carry procedural risk, require specialist infrastructure, have strict eligibility criteria, and are declined by a meaningful proportion of patients [3]. Among pharmacological alternatives, propranolol is an adjunctive symptomatic option, while clonazepam and anticholinergics help some patients but bring sedative, cognitive, and autonomic adverse effects that are frequently poorly tolerated in older individuals, limiting their evidence-supported role. BoNT, already central to the management of focal dystonias and cervical dystonia, represents a targeted and reversible off-label option whose role in medication-refractory PD tremor is the subject of this review.

The intellectual objection is straightforward: PD rest tremor is generated by pathological oscillations within the basal ganglia–thalamo-cortical circuit, not by intrinsically abnormal peripheral muscle activity. Treating a central tremor with a peripheral neuromuscular blocking agent seems, on the face of it, to be targeting the symptom rather than the mechanism. Whether that objection holds up under neurophysiological and clinical scrutiny is what this review examines. Studies were identified by PubMed search (botulinum toxin AND Parkinson’s disease AND tremor; botulinum toxin AND tremor; condition-specific supplementary searches) covering 1990 to January 2024, prioritizing randomized controlled trials and then prospective series. Where recommendations go beyond controlled trial evidence, they are stated and labeled as expert opinion.

## 2. Pathophysiology: Does Peripheral BoNT Have Any Business in a Central Tremor?

PD rest tremor originates from pathological 4 to 6 Hz oscillations within the basal ganglia, amplified through the cerebello-thalamo-cortical circuit [4,5,6]. The peripheral muscles are not generating the tremor; they are expressing it. Levodopa and DBS work because they act on the generator. BoNT acts at the neuromuscular junction, as far downstream as one can get. The question is whether downstream action has any upstream consequence.

There is a credible mechanism by which it might. BoNT cleaves SNAP-25, blocking acetylcholine release at the NMJ and reducing extrafusal contraction. Less appreciated is its effect on the gamma motor system: reduced intrafusal muscle fiber contraction decreases muscle spindle sensitivity and diminishes Ia afferent firing [7]. Since Ia afferents carry proprioceptive signals back to the cerebellum and sensorimotor cortex, reduced Ia input may partially de-entrain the central oscillatory loop. This is not pharmacological speculation. TMS measurements before and after individualized EMG-guided BoNT injection showed significant post-injection reductions in intracortical facilitation and increases in intracortical inhibition in both PD tremor patients [8], a pattern consistent with centrally mediated effects driven by reduced peripheral afferent input. A scoping review of BoNT’s effect on sensorimotor integration across PD tremor, cervical dystonia, and writer’s cramp found convergent evidence that repeated injections modify cortical circuits beyond the simple reduction in muscle activity [9].

None of this means BoNT is equivalent to DBS in PD tremor or that peripheral injection addresses the core pathology. It does mean that the objection ‘this is a central tremor; peripheral injection is irrelevant’ is an oversimplification. The peripheral and central nervous systems communicate continuously, and modifying the afferent limb of that communication has measurable central effects. Whether those effects are clinically meaningful is a question for the trial data. Where mechanistic evidence is drawn from non-PD conditions such as essential tremor, cervical dystonia, or writer’s cramp, this is an extrapolation and is identified as such; PD-specific mechanistic data remain limited.

## 3. Clinical Evidence

### 3.1. BoNT in PD Tremor

Early open-label series through the 1990s and 2000s reported tremor reduction in PD patients using EMG-guided injection, but the sample sizes were small, methodology varied considerably, and none were controlled [10,11]. The field moved forward when a double-blind, placebo-controlled crossover RCT enrolled 30 patients with medication-refractory PD rest tremor [12]. An EMG needle was used to screen up to 14 forearm and arm muscles at rest and with the arm outstretched; only muscles with 4–6 Hz tremor-frequency discharge were injected, with the dose individualized to EMG amplitude and estimated muscle bulk (mean 100 U IncoA, range 85–110 U, via 7–12 injections per patient). UPDRS rest tremor subscores (the primary endpoint) improved significantly versus placebo at four and eight weeks (*p* < 0.001), with significant gains also in action/postural tremor at eight weeks (*p* = 0.01) and in patient-perceived change on the Patient Global Impression of Change at four and eight weeks (*p* < 0.001). Grip strength did not differ significantly from placebo (*p* = 0.32), and disabling hand weakness occurred in 6.6% of patients, a rate that would prove important when set against the 30–70% reported with fixed-dose approaches.

A retrospective series from Baylor College of Medicine spanning 2010–2018 included 91 patients with refractory hand tremor of various etiologies, of whom six had PD [13]. OnabotulinumtoxinA was injected individually into forearm flexors, with EMG guidance used in only 5.5% of sessions. Moderate or marked improvement (≥3/4 on a peak-effect scale) was reported in 80–86% of patients across tremor types at first and last visit; the response profile was sustained over a mean 29.6 months and 7.7 injection sessions per patient. Weakness was transient and non-disabling in 12.2% of 1095 limb injection sessions. The six PD patients were not reported separately, but the real-world durability and safety profile across this large series supports the practical viability of individualized forearm BoNT for refractory tremor.

A 2025 crossover RCT of EMG-guided IncoA for proximal upper limb tremor enrolled 20 patients and found significant improvement on clinician-rated scales (TETRAS, FTM-TRS) but a negative result on the pre-specified primary endpoint, the patient-centered Goal Attainment Rating Scale [14]. This trial warrants mention but not emphasis in the PD context: of the 20 patients enrolled, only one had PD. The cohort comprised predominantly essential tremor and essential tremor plus, and the findings cannot be extrapolated to PD-specific practice. The trial’s main contribution is methodological, demonstrating that clinician-rated tremor improvement does not reliably translate into patient-perceived functional benefit.

Open-label kinematic-guided series by Samotus and Jog tracked PD patients over 48–96 weeks using sensor-based biomechanical dosing [15,16]. UPDRS rest tremor scores improved consistently at 16, 32, and 48 weeks. The mean dose was substantially higher than in the pivotal RCT series (174 ± 69 U IncoA), reflecting the kinematic protocol’s inclusion of more proximal muscles. A 34% withdrawal rate due to weakness or insufficient benefit is notable and reflects the technical demands of the approach. The PD-specific evidence base currently consists of one positive DB-RCT [12] and supportive open-label data [13,15,16,17]. That is a thin evidence base by the standards of an established therapy. All the studies are summarized in Table 1. 

### 3.2. BoNT Across Other Tremor Conditions

The non-PD tremor literature is larger in aggregate but equally heterogeneous. It is reviewed here because the technical and conceptual lessons it contains apply directly to PD practice and because the weakness rates and outcome patterns across conditions set the quantitative context that PD-specific evidence alone cannot provide. The key findings are summarized in Table 2.

Essential tremor has been the most extensively studied. The first two RCTs used a fixed-dose, fixed-muscle approach, predetermined forearm wrist flexors and extensors, uniform doses, and no individualization [18,19]. Both showed tremor scale improvement; both showed hand weakness in 30–70% of patients, with no consistent functional benefit. These are not historical curiosities. They remain the most informative data on what happens when technique is wrong, and they provide the quantitative benchmark against which individualized approaches should be measured. The two subsequent RCTs used individualized EMG-guided [20] and kinematic-guided [21] injection, respectively, achieving equivalent tremor reduction with weakness in fewer than 15% of patients. The 2025 ELATE Phase 2 trial (n = 182, multicenter DB-RCT) has been reported in conference/topline form to have met its primary disability-scale endpoint, with weakness in 24.5% versus 2.3% for placebo [22]. As these results are preliminary and not yet available as a peer-reviewed full report, ELATE is described here as the largest BoNT tremor trial to date but is not assigned definitive Class I status pending full publication and regulatory evaluation. A recent meta-analysis found moderate patient-reported benefit but no statistically significant improvement in clinician-rated tremor severity across pooled post-intervention scores and no grip strength reduction [23].

Head tremor received its highest-quality evidence from a 117-patient French multicenter DB-RCT of bilateral splenius capitis injection at weeks 0 and 12 [24]. The primary endpoint, a clinically meaningful improvement on the CGI-C at week 18, was met (31% vs. 9%; *p* = 0.009), and quality of life improved. Adverse events were substantially more frequent with BoNT (47% vs. 16%), including one patient requiring hospitalization for severe dysphagia. Benefit waned by week 24 in some patients, consistent with the pharmacokinetics of BoNT. This trial matters to the PD field not because head tremor and PD tremor share a mechanism but because it demonstrates what a properly powered, technique-standardized, disability-primary tremor RCT can achieve.

For dystonic hand tremor, a DB-RCT of 30 patients [25] used EMG-guided individualized OnabotulinumtoxinA (mean six muscles, 63 U mean dose) and showed significant improvement at 6 and 12 weeks with no statistically significant grip weakness [25]. This is the cleanest demonstration in the tremor–BoNT literature that individualized technique achieves Class I evidence without a meaningful weakness penalty.

MS-related upper limb tremor, predominantly cerebellar in character, showed significant improvement in a DB-RCT of 33 upper limbs [26], but weakness occurred in 42% of treated limbs. The lesson for PD is specific: when a patient already has compromised hand function from bradykinesia, the acceptable weakness window narrows considerably, and doses must be correspondingly conservative. Boonstra et al. subsequently demonstrated fMRI evidence of BoNT-induced changes in sensorimotor integration cortical regions in MS tremor patients [27], adding to the central-effect data alongside the TMS findings in PD [8].

Voice tremor responds to EMG-guided thyroarytenoid injection with 50–65% subjective improvement, and the limited adverse effects (transient breathiness, mild dysphagia) resolve within 1–2 weeks [28]. Primary writing tremor and task-specific tremor in musicians respond to very small doses in very few muscles [29,30]. Orthostatic tremor did not respond in the only controlled trial [31], and this negative Class I result is the only firm ‘BoNT does not work’ finding in the tremor literature. Holmes, palatal, chin, and jaw tremors have case-series evidence with variable results [32,33,34,35].

Three points from this cross-condition overview apply directly to PD practice, with explicit acknowledgment of where the analogy has limits. First, the technical finding transfers across conditions, though not without qualification: Individualized EMG-guided injection has reduced weakness from 30 to 70 percent to below 15 percent in the conditions studied while maintaining efficacy, although the magnitude of this reduction and the residual weakness risk vary with the tremor syndrome, the muscles injected, baseline motor impairment, dose, and guidance method. Second, the methodological finding is also transferable: The consistent dissociation between clinician-rated tremor improvement and patient-perceived benefit [19,23] reflects how BoNT affects tremor scales versus lived function generally, not just in one condition. Patient-centered co-primary endpoints are required in future trials. Third, the inference that non-PD RCTs support PD practice requires caution: PD tremor has a distinct central pathophysiology, and non-PD evidence supports the technical approach and provides safety benchmarks rather than substituting for PD-specific trial data. In all conditions studied, BoNT benefits last 8 to 12 weeks, establishing cyclical repeat injection as the required clinical model.

**Table 2:** BoNT evidence across tremor conditions: summary and transferable lessons for PD practice.

Condition	Best Evidence	No of Patients (n)	Key Result	Weakness	Class	PD Lesson
PD tremor	Mittal 2017 [12]	30	UPDRS rest tremor sig. reduced at 4 & 8 weeks; individualized technique	6.7%	I	One positive DB-RCT; distal focus; proof of concept
Essential tremor	Jog 2020 [21] (individualized EMG/kinematic);	87	Individualized: weakness < 15%;	<15% (individualized)	I	Fixed-dose failure (30–70% weakness)
	ELATE 2025 [22] (conference/topline)	182	ELATE met disability primary endpoint	24.5%	ELATE topline	Technique is the determinant
Head tremor	Marques 2023 [24]	117	CGI-C primary met (31% vs. 9%); QoL improved; 47% AEs including severe dysphagia	47% AEs	I	Largest tremor DB-RCT; sets power and design standard
Dystonic tremor	Rajan 2021 [25]	30	Sig. improvement 6 & 12 weeks; individualized EMG-guided; no significant grip weakness	NS	I	Best tolerability data; technique eliminates weakness penalty
MS tremor	Van der Walt 2012 [26]	33 limbs	Sig. tremor improvement; fMRI central effect (Boonstra 2020 [27])	42%	III	Pre-existing weakness narrows acceptable window; mirrors PD bradykinesia
Voice tremor	Pahwa 1995 [28]	10	50–65% subjective improvement; transient AEs; distinct laryngeal mechanism	Transient dysphonia	III	Relevant in co-occurring PD voice tremor
Orthostatic tremor	Bertram 2013 [31]	7	No benefit; tibialis anterior injection	Minimal	I (negative)	Only Class I negative result in tremor–BoNT literature

DB-RCT: double-blind randomized controlled trial; NS: not significant; AEs: adverse events; CGI-C: Clinical Global Impression of Change; QoL: quality of life; MS: multiple sclerosis.

## 4. Injection Technique

### 4.1. Why Fixed-Dose Fixed-Muscle Injection Does Not Work

The evidence here is unambiguous. Fixed-dose injection of forearm flexors and extensors at predetermined doses, the approach used in the first two ET RCTs, produced dose-dependent tremor scale improvement but hand weakness in 30% of patients at low doses and 70% at high doses, with no consistent functional benefit [18,19]. The mechanism is now quantifiable: excluded-coherence analysis shows that the dominant tremor-driving muscle varies substantially between individuals [36], so a fixed template will systematically inject non-driving muscles (causing weakness) while potentially under-injecting the muscle actually responsible. This is not a simplified version of individualized injection. On the available evidence it is an inadequate approach that should be avoided whenever individualized, EMG- or kinematic-guided injection is feasible.

### 4.2. EMG-Guided Individualized Injection

The technique is straightforward in principle, though it requires training and EMG equipment. An EMG needle is used to sample 8–14 forearm and arm muscles sequentially, at rest with the arm supported and then with the arm outstretched. Any muscle showing 4–6 Hz tremor-frequency discharge is a candidate for injection; any muscle that is electrically silent is left alone. The dose in each injected muscle is calibrated to the amplitude of the tremor discharge and the estimated bulk of the muscle. Total doses of 80–100 U IncoA across 9–12 muscles per limb are typical in PD tremor. The session takes 30–45 min in experienced hands. The pivotal PD tremor RCT used this approach and achieved 6.7% moderate weakness [12]; the individualized EMG-guided ET RCT [20] achieved comparable tremor reduction with fewer than 10% of patients experiencing weakness. The technique is reproducible in a movement disorders clinic with standard EMG equipment.

### 4.3. Kinematic Sensor-Guided Protocol

The Western University approach places inertial sensors at the wrist, forearm, elbow, and upper arm to decompose the tremor into its directional components at each joint and guide both muscle selection and dose. It is more precise in theory but considerably more resource-intensive in practice, requiring proprietary sensors and analysis software. Open-label PD data show sustained improvement over 96 weeks, but the 34% withdrawal rate due to weakness or insufficient benefit [15,16] indicates that additional precision does not automatically translate to superior outcomes when more proximal muscles are included and doses are higher. Both techniques are superior to fixed injection; the EMG-guided approach is more widely replicable given its reliance on standard clinic equipment.

### 4.4. Ultrasound Guidance

Ultrasound guidance is preferred whenever the equipment is available. When a sonography device is present, injecting under direct ultrasound visualization is recommended for both superficial and deep muscles, since real-time needle visualization, confirmation of intramuscular placement, and pre- and post-injection tremor amplitude measurement are useful at every target. Where ultrasound is not available, EMG-confirmed needle placement is an acceptable alternative for superficial forearm muscles. For deep muscles—supinator (adjacent to the posterior interosseous nerve), flexor digitorum profundus, and flexor pollicis longus (adjacent to the median nerve and radial artery)—ultrasound guidance is strongly recommended on anatomical-safety grounds, irrespective of whether it is used routinely for other muscles [37]. This recommendation reflects anatomical safety considerations and expert opinion rather than tremor-specific comparative trial evidence.

## 5. Muscle Selection and Dosing

Muscle selection is guided by the EMG screen, not by anatomy. The practical hierarchy is as follows: pronator teres first (drives the rotational pill-rolling component; weakness of pronation is well tolerated); flexor carpi radialis (FCR) and flexor carpi ulnaris (FCU) in every first cycle (the core wrist flexor targets); low-dose FDS, 5 to 10 U incobotulinumtoxinA, only if the finger-rolling component persists after adequate wrist and pronator injection; lumbricals from the palmar surface (5 to 10 U) for the finger-adduction component of pill-rolling; and flexor pollicis longus (FPL) for persistent thumb-to-index opposition, with ultrasound/EMG guidance strongly recommended given its proximity to the median nerve and radial artery. FDP is rarely needed and should not be co-injected with FDS at meaningful doses. The total finger flexor dose should not exceed 30 U incobotulinumtoxinA in a first cycle. Wrist and finger extensors, ECR, ECU, and EDC, must not appear in a routine injection template. Extensor weakness produces wrist drop and finger drop. These are not acceptable side effects. They are entirely preventable by confining injection to the muscles actually driving the tremor.

## 6. Patient Selection, Preparation, and Follow-Up

### 6.1. Who Is a Reasonable Candidate

BoNT is appropriate for PD patients with tremor that is functionally disabling despite optimized dopaminergic therapy and is predominantly distal (wrist, finger, or re-emergent pattern) and where DBS and FUS have been discussed and are unavailable, contraindicated, or declined after adequate information. Pre-existing hand weakness from any cause is a relative contraindication. Patients with akinetic-rigid PD and minimal tremor are not candidates. The treating center must be able to offer sonography and/or EMG-guided individualized injection; fixed-dose injection without muscle localization is not an acceptable substitute for this indication.

Off-label status must be disclosed and documented before any injection. Informed consent should specifically address the nature and expected duration of weakness, the off-label status of the treatment, and the expected duration of benefit (8–12 weeks requiring repeat injection).

### 6.2. Preparation

IncobotulinumtoxinA is the only preparation studied in a double-blind RCT for PD tremor [12]; for that reason, it is the formulation with the most direct PD-specific evidence, rather than an inherently preferred product. Its freedom from complexing proteins has been proposed to reduce immunogenic neutralizing antibody formation with repeated injection, which would be relevant given that PD tremor requires indefinite cyclical treatment, although this is a pharmacological rationale rather than a demonstrated clinical advantage in this indication. OnabotulinumtoxinA is approximately dose-equivalent (1:1) and is supported by the broadest historical tremor experience, including the 2025 ELATE trial reported in conference/topline form [22]. AbobotulinumtoxinA requires 2.5–3 times the IncoA/OnaA units. Applying IncoA doses to an AboA prescription is a medication error that can cause severe overdose-related weakness; this must be explicit in any institutional protocol. In practice, product selection should follow local availability, clinician experience, dosing familiarity, and regulatory constraints.

### 6.3. Follow-Up and Outcome Assessment

Response to BoNT injection typically becomes apparent within two to four weeks and reaches its peak at four to eight weeks. This is the optimal window for clinical reassessment. At that visit, record the UPDRS part III tremor subscore or TETRAS, grip dynamometry, and the patient’s own assessment of whether the specific activities that brought them to treatment have improved. Scale improvement without patient-perceived functional benefit is not a successful outcome. If two full cycles produce no patient-reported benefit, the therapy should be reconsidered rather than continued on the basis of rating scale change alone. Muscle selection and doses are adjusted at each cycle based on the preceding response and weakness profile. 

## 7. Critical Appraisal: What the Evidence Does and Does Not Support

The PD-specific evidence consists of one positive DB-RCT of 30 patients [12] and supportive open-label data [13,15,16]. The Nagaratnam 2025 trial [14] enrolled only one PD patient and is not PD-specific evidence, though its finding of a negative primary patient-centered endpoint despite significant clinician-rated tremor improvement is a methodologically important signal. Against the five ET RCTs culminating in ELATE [22] and the 117-patient head tremor NEJM trial [24], the PD evidence base is underpowered and unconfirmed by independent replication. A positive RCT in 30 patients with individualized technique and 6.7% weakness is a meaningful signal; it is not sufficient to establish BoNT as a routinely offered therapy.

What the evidence supports, with appropriate qualification: In a carefully selected patient with medication-refractory distal PD tremor, treated by a trained clinician using individualized EMG-guided injection, incobotulinumtoxinA at 80 to 100 U per limb across EMG-confirmed active muscles can reduce UPDRS rest tremor scores at four to eight weeks, with a weakness rate below 10 percent in experienced hands [12]. The effect is time-limited to eight to twelve weeks and repeatable without evidence of tachyphylaxis [15,16]. The technique is inseparable from the efficacy claim: the positive RCT used individualized injection, and the tolerability data derive from individualized injection. Outcomes at specialist centers should not be assumed to generalize to settings without equivalent training and case volume.

What the evidence does not support: BoNT before pharmacological optimization is genuinely complete; fixed-dose injection; treatment by clinicians without specific EMG training in tremor assessment; or treating clinician-rated scale improvement as proof of treatment benefit without independently assessing patient-reported function. Evidence from ET, dystonic tremor, and head tremor RCTs informs the technical approach and safety expectations; it does not establish PD tremor efficacy independently.

Based on current US and EU prescribing information for onabotulinumtoxinA and incobotulinumtoxinA, neither product lists PD tremor among its approved indications; BoNT is therefore used off-label for this indication, and we are not aware of any jurisdiction in which it is approved for PD tremor. Off-label use is defensible when the patient has been fully informed of the evidence base, its limitations, the regulatory status, the expected duration of benefit, the requirement for repeat injection, and the specific risks, including weakness, wrist drop, and finger drop. It is not defensible when offered as equivalent to licensed therapies, when the technique prerequisites are absent, or when the clinician treats scale improvement as sufficient evidence of benefit. The off-label status of this therapy is a genuine statement about the current limits of what is known, not a bureaucratic formality.

## 8. A Practical Step-by-Step Injection Protocol

The following protocol synthesizes the individualized EMG-guided approach validated in controlled trials [12,20] with the kinematic assessment framework developed by Samotus and Jog [15,16,38] and the clinical guidance of Mittal and Pandey [39] and Mittal, Lenka and Jankovic [39]. It is an expert-opinion implementation framework derived from limited PD-specific trial evidence and extrapolation from other tremor syndromes, not a validated guideline or a rigid algorithm. It is intended as an operational guide for the clinician performing these injections, and clinical judgment governs every step.

**Step 1. Clinical Assessment of Tremor Phenomenology**

Before any EMG or instrument is used, the tremor must be characterized clinically across all relevant postures. Examine the patient with the arm at rest supported on the lap, then with the arm outstretched and unsupported, then during action (finger-to-nose, pouring water between cups, writing, drawing an Archimedes spiral). Identify the dominant movement components: Is the tremor predominantly rotational at the forearm (pronation–supination, classic pill-rolling)? Is there a wrist flexion–extension oscillation? Is the finger flexion–adduction component the most functionally limiting feature? Is the thumb separately involved? Does the tremor re-emerge after a brief latency when the arm is outstretched (re-emergent tremor), which localizes differently from pure rest tremor? Each of these patterns implicates a different primary muscle set and guides where the EMG screen should begin [39,40].

Assess the joints in sequence: shoulder (abduction–adduction oscillation at rest), elbow (flexion–extension arc during action), forearm (pronation–supination rotation, the most characteristic PD movement), wrist (flexion–extension or radial–ulnar deviation), and fingers (pill-rolling index–thumb opposition, individual finger flexion oscillation). This joint-by-joint inspection maps to the muscle screen sequence in Step 2. Document the dominant component: most PD patients have a forearm rotation and wrist flexion pattern; pure elbow or shoulder oscillation as the sole complaint is uncommon and warrants re-evaluation of the diagnosis.

**Step 2. EMG Screening**

The EMG screen is the core of the individualized approach and the step that most distinguishes it from fixed injection [12,38]. With the patient seated and the arm resting supported, insert an EMG needle into each candidate muscle and record activity at rest. Then ask the patient to extend the arm fully outstretched and record again. The tremor-frequency discharge of 4 to 6 Hz will be visible as rhythmic bursting on the EMG trace. Only muscles showing this discharge are injection candidates. Muscles that are electrically silent are not injected regardless of anatomical proximity to the tremor movement.

Screen in the following sequence, guided by the clinical assessment in Step 1: Begin in the forearm: The pronator teres is the first muscle to sample in any patient with a rotational component. Then screen the flexor carpi radialis and flexor carpi ulnaris. If the patient has finger involvement, screen the flexor digitorum superficialis. If thumb rolling is present and forearm flexor injection has not previously resolved it, screen the flexor pollicis longus using ultrasound guidance (see Step 3). At the elbow, screen the biceps brachii and brachioradialis if there is a flexion arc component. At the shoulder, screen the deltoid only for severe proximal rest tremor that has failed distal injection. Screen 8 to 14 muscles in total across both rest and outstretched positions [12,38]. Do not inject any muscle not screened. Do not inject any muscle that is silent on EMG.

The kinematic alternative, for centers with access to the required equipment, places inertial sensors at the wrist, forearm, elbow, and upper arm and records tremor direction and amplitude at each joint during standardized postures. This provides a quantitative biomechanical map of which joints contribute most to the tremor displacement and in which direction, guiding muscle selection and relative dose allocation across segments [15,16]. The kinematic approach adds precision but increases resource demands and session duration considerably. For most movement disorder clinics, the EMG-guided approach is more practical and has stronger controlled trial evidence in PD tremor specifically [12].

**Step 3. Muscle Localization and Injection Guidance**

Ultrasound guidance is preferred for all muscles whenever the equipment is available. When a sonography device is present, injecting every muscle under direct ultrasound visualization, regardless of depth, is the preferred approach. Where ultrasound is not available, surface anatomy and EMG-confirmed needle placement are recommended. Pronator teres is accessible 4 to 6 cm distal to the medial epicondyle on the volar forearm. FCR and FCU lie in the proximal and mid-volar forearm, respectively; inject FCR 5 to 7 cm proximal to the wrist crease (avoiding the radial artery) and FCU just medial to the FCR tendon (avoiding the ulnar nerve at the ulnar groove). FDS is accessible in the mid-forearm between the FCR and FCU compartments.

The supinator lies deep in the lateral forearm, immediately adjacent to the posterior interosseous nerve (PIN); blind or EMG-only injection carries a meaningful risk of PIN injury and should be performed carefully. Flexor digitorum profundus is deep to FDS and best visualized with ultrasound before injection. Flexor pollicis longus runs in the deep volar forearm between the radial artery laterally and the median nerve medially; the injection target is the mid-forearm belly, confirmed under direct ultrasound visualization. Ultrasound also enables pre-injection measurement of tremor frequency and amplitude within the target muscle, which can be compared at follow-up as an objective within-session response marker [37].

**Step 4. Dose Selection**

Dose is individualized to three factors: the amplitude of the EMG tremor discharge in that muscle (higher amplitude warrants a higher dose), the estimated bulk of the muscle (larger muscles tolerate higher doses before producing weakness), and the clinical priority of that muscle based on the Step 1 assessment. The principle is to use the minimum dose that produces a meaningful reduction in tremor-frequency discharge, not the maximum tolerated dose. Starting doses are shown in Table 3; these are first-cycle doses for a treatment-naive patient. For subsequent cycles, titrate upward in muscles where tremor reduction was insufficient and weakness was absent and reduce it in muscles where weakness occurred [38,39].

The guiding hierarchy for dose allocation across muscles is as follows: The pronator teres receives the full starting dose if active on EMG (20 to 30 U IncoA); FCR and FCU similarly receive their standard doses (15 to 20 U each) if active. FDS, if included, is started at the low end of the range (5 to 10 U) and increased only if the first cycle produces no finger weakness and inadequate finger tremor suppression. The total first-cycle dose per limb should not routinely exceed 100 U of IncoA. Higher doses are possible in subsequent cycles for patients with adequate tolerability and persistent tremor, but each dose increase requires explicit review of the benefit–weakness balance at the preceding cycle [12,16].

**Step 5. Injection Execution**

Inject under ultrasound guidance whenever the equipment is available; this is the preferred approach regardless of muscle depth. Ultrasound confirms intramuscular needle placement, allows real-time visualization during injection, and enables pre- and post-injection tremor frequency measurement as an objective within-session response marker. Where ultrasound is not available, proceed with EMG-confirmed placement for superficial muscles. Reconstitute incobotulinumtoxinA/onabotulinumtoxinA with 1 mL of preservative-free normal saline in 100 units to obtain 100 U/mL; adjust concentration based on dose range and muscle bulk. Use a 27-gauge or finer needle. Confirm EMG position by recording tremor discharge immediately before injecting, then inject slowly. Record each muscle injected, the dose, and the EMG findings; this record is essential for adjusting the next cycle.

**Step 6. Reassessment and Cycle Adjustment**

Review the patient at 4 to 8 weeks, which corresponds to the peak BoNT effect. Assess two domains independently. First, clinician-rated tremor: score the UPDRS part III tremor items (items 15 to 18) or TETRAS subscore and compare them with the pre-injection baseline. Second, patient-reported functional benefit: ask the patient directly whether tremor interference with their specific functional goals (identified before the first injection) has improved. Scale improvement without patient-perceived functional benefit does not constitute treatment success. Assess grip strength with a dynamometer and ask specifically about functional weakness in daily tasks (opening jars, lifting cups, buttoning). Document all four: tremor rating, patient goal attainment, grip strength, and reported weakness.

Based on this assessment, adjust the next cycle as follows: If tremor is reduced and there is no meaningful weakness, maintain the same muscle set and doses for the next injection at 12 to 16 weeks. If tremor is reduced but weakness is present in a specific muscle, reduce the dose in that muscle by 20 to 30 percent or omit it entirely, depending on severity. If tremor reduction is inadequate in a specific component and there was no weakness, increase the dose in the relevant muscle or add an adjacent muscle if not previously screened. If there is neither tremor reduction nor patient benefit after two complete cycles with optimized doses, discontinue BoNT and reconsider the overall management approach, including candidacy for DBS or FUS [38,39].

## 9. What the Field Needs

The field requires a multicenter DB-RCT in PD tremor specifically, with co-primary endpoints of a validated tremor rating scale and a patient-centered functional outcome measure (GARS or equivalent) and a standardized EMG-guided injection protocol. Any sample-size target should be justified by a formal power calculation specifying the anticipated effect size, the primary outcome and its minimal clinically important difference, alpha, power, anticipated dropout, and whether a parallel or crossover design is used, rather than asserted a priori. The ELATE [22] and Marques [24] trials demonstrate that this is achievable. Beyond the pivotal trial: prospective characterization of the learning curve across centers; direct comparison of EMG-guided and kinematic techniques in the same RCT; neurophysiological predictors of response (TMS, excluded-coherence analysis); and a formal safety registry tracking weakness, dose, and functional outcomes across repeated injection cycles.

## 10. Limitations

The PD-specific evidence base rests on a single DB-RCT of 30 patients that has not been independently replicated [12]. The trial was conducted at a specialist center with considerable injection expertise; outcomes should not be assumed to transfer to settings without equivalent training and infrastructure. Non-PD tremor evidence is incorporated as indirect support for technique and safety benchmarks, but PD tremor has a distinct central pathophysiology, and the response profile may differ. The injection protocol in Section 9 is expert opinion synthesized from published techniques; it is not a validated guideline. Dose ranges reflect published series and expert consensus, not a dedicated dose-finding trial for PD tremor. Unit equivalence between preparations follows general conventions that are not formally validated for this indication. As an off-label therapy in all jurisdictions, the risk–benefit assessment for individual patients must account for the absence of regulatory review and the absence of post-marketing pharmacovigilance data specific to this indication.

## 11. Conclusions

Tremor-dominant PD refractory to medication is a common clinical problem with limited pharmacological solutions. BoNT offers a mechanistically plausible and technically feasible option for patients in whom DBS and FUS are not available or acceptable, provided that individualized EMG-guided injection is used. The evidence for this is real but narrow: one positive double-blinded randomized controlled trial of 30 patients without independent replication, open-label series, and a body of non-PD tremor data that informs technique rather than establishing PD-specific efficacy. The critical practical messages from this evidence are few and consistent: select patients carefully; avoid extensors; start with pronator teres, flexor carpi radialis, and flexor carpi ulnaris; keep doses conservative in the first cycle; and measure what the patient experiences, not just what the clinician rates. It must also be acknowledged that the careful patient selection this approach depends on, while it helps the individual clinician and patient achieve tremor relief, works against assembling a large, statistically representative cohort: such selection departs from the unselected real-world PD population and introduces selection bias that constrains generalizability. A well-conducted multicenter randomized controlled trial with patient-centered co-primary endpoints, designed to mitigate this selection bias, remains the field’s most important unmet need. Until that trial exists, BoNT for PD tremor is a specialist option, not a standard therapy, and should be offered and described accordingly.

## 12. Materials and Methods

This narrative critical review searched PubMed, Embase, and Cochrane CENTRAL for articles published between January 1990 and January 2024, combining “botulinum toxin” (and “botulinum neurotoxin,” “BoNT”) with “tremor,” “Parkinson’s disease tremor,” “essential tremor,” “dystonic tremor,” “multiple sclerosis tremor,” “head tremor,” “voice tremor,” and “orthostatic tremor.” As a narrative rather than systematic review, no formal selection flow diagram or duplicate independent screening was performed.

Randomized controlled trials were prioritized, followed by prospective then retrospective series, and studies of tremor of any etiology were included where their technical, safety, or outcome findings were relevant to Parkinson’s disease tremor, distinguishing throughout between PD-specific and extrapolated evidence. Where studies are referred to by evidence class, classification follows the American Academy of Neurology scheme for therapeutic studies [41]: Class I denotes an adequately powered, prospective, randomized controlled trial with masked or objective outcome assessment, concealed allocation, and clearly defined primary outcomes; Class II denotes an RCT lacking one of these criteria or a matched prospective cohort; and Class III denotes other controlled or uncontrolled studies, including open-label and retrospective series.

Outcomes were extracted as reported by the original authors using their own scales (UPDRS, TETRAS, Fahn–Tolosa–Marín Tremor Rating Scale, Goal Attainment Rating Scale, Clinical Global Impression of Change, grip dynamometry, and reported weakness rates); because these scales are not directly comparable, results are presented descriptively rather than pooled, and no quantitative meta-analysis was performed. Recommendations that extend beyond controlled trial evidence, including the step-by-step protocol and the muscle and dose selections, are explicitly identified as expert opinion.

## Figures and Tables

**Table 1:** Key controlled studies and real-world series in PD and proximal upper limb tremor.

Reference	Design	Preparation & Dose	Technique	Primary Outcome	Weakness	Class
Mittal et al. 2017 [12]	DB-RCT crossover n = 30, PD rest tremor	IncoA 80–100 U/limb	EMG-guided 8–12 muscles	UPDRS rest tremor at 4 & 8 weeks	6.7% moderate (extensors)	Class I
Nagaratnam et al. 2025 [14]	DB-RCT crossover n = 20 proximal UL tremor (only 1/20 with PD; not PD-specific)	IncoA 180 ± 60 U/limb	EMG-guided proximal focus	GARS (patient-centered primary): negative TETRAS/FTM-TRS: sig.	Not disabling	Class I (negative primary; not PD-specific)
Samotus et al. 2017, 2020 [15,16]	Open-label n = 20 PD 96 weeks	IncoA 174 ± 69 U/limb	Kinematic sensors (Western U.)	UPDRS rest tremor sustained at 16, 32, 48 weeks	34% withdrawal (weakness or lack of benefit)	Class III
Niemann & Jankovic 2018 [13]	Retrospective series n = 91 (6 PD) 29.6 months follow-up	OnaA 65–79 U/limb (increasing over time)	Individualized FF; EMG in 5.5% of sessions	80–86% moderate/marked peak-effect rating; stable over sessions	12.2% of sessions; transient, non-disabling (mean 36 days)	Class III

n—number of patients; DB-RCT: double-blind randomized controlled trial; IncoA: incobotulinumtoxinA; OnaA: onabotulinumtoxinA; UL: upper limb; UPDRS: Unified Parkinson’s Disease Rating Scale; GARS: Goal Attainment Rating Scale; TETRAS: Essential Tremor Rating Assessment Scale; FTM-TRS: Fahn–Tolosa–Marín Tremor Rating Scale; FF: forearm flexors. Note: Nagaratnam 2025 [14] enrolled only 1/20 patients with PD and should not be classified as PD-specific evidence.

**Table 3:** Muscle injection guide for BoNT in PD tremor.

Segment	Muscle	Movement	IncoA/OnaA	AboA	Clinical Note
Shoulder	Deltoid (middle head)	Abduction	20–40 U	55–100 U	Reserve for severe proximal rest tremor when distal injection alone has failed. Arm elevation weakness is immediately and severely disabling; discuss explicitly and document consent before injecting.
Elbow	Biceps brachii	Flexion	15–30 U	40–80 U	Confirm EMG activity before injecting. Impairs lifting, carrying, and elbow flexion against gravity. Brachioradialis preferred when tremor localizes with forearm in neutral position.
	Brachioradialis	Flexion/neutral rotation	20–30 U	55–80 U	Preferred over biceps for neutral-forearm flexion tremor. More accessible to EMG needle. Weakness of elbow flexion is generally better tolerated than biceps weakness.
	Triceps brachii (long head)	Extension	10–30 U	30–80 U	Rarely the primary driver in PD tremor. Screen only if patient reports elbow extension oscillation. Impairs pushing and overhead reach. Confirm EMG before injecting.
Forearm	Pronator teres	Pronation	10–30 U	30–80 U	Highest-yield single muscle in PD pill-rolling tremor. Screen first in every patient with a rotational component. Drives pronation–supination oscillation. Pronation weakness is well tolerated in daily activities.
	Supinator	Supination	15–25 U	40–65 U	Deep muscle; ultrasound guidance mandatory to avoid posterior interosseous nerve (PIN). Inject only if supination-dominant tremor is confirmed on EMG.
Wrist	Flexor carpi radialis (FCR)	Wrist flexion	15–20 U	40–55 U	Core EMG protocol target. Inject 5 cm proximal to the wrist crease. Avoid the radial artery distally. Screen in every patient with wrist tremor.
	Flexor carpi ulnaris (FCU)	Wrist flexion	15–20 U	40–55 U	Core EMG protocol target. Screen separately from FCR, as FCU may have independent tremor-frequency discharge. Avoid the ulnar nerve at the ulnar groove.
	ECR longus/brevis	Wrist extension	Avoid routinely	—	WRIST DROP RISK. Extensor weakness produces wrist drop: inability to extend the wrist against gravity. Do not include in routine templates. Use only if EMG confirms dominant extensor tremor with no flexor activity, and only with explicit patient consent for wrist drop risk specifically.
	Extensor carpi ulnaris (ECU)	Wrist extension/ ulnar deviation	Avoid routinely	—	WRIST DROP RISK. Same principle as ECR. Do not include in routine templates. Use only if ulnar deviation-dominant tremor is confirmed on EMG with no flexor activity present.
Fingers	Flexor digitorum superficialis (FDS)	Finger flexion	5–15 U	15–45 U	Add only if finger-rolling component persists after adequate wrist and pronator injection. Start at low dose (5–10 U IncoA); grip weakness is uncommon at this range. Do not co-inject FDS and FDP at meaningful doses simultaneously.
	Flexor digitorum profundus (FDP)	Finger flexion	5–10 U (rarely needed)	15–25 U	Use only if FDS injection alone has not controlled the finger component and EMG confirms active FDP discharge. Ultrasound guidance strongly recommended. Never co-inject FDP and FDS at full dose.
	Flexor pollicis longus (FPL)—PD-specific	Thumb flexion	5–15 U	15–40 U	PD-SPECIFIC. Drives thumb opposition in pill-rolling. Absent from most published templates. Clinical indication: persistent rhythmic thumb-to-index apposition after adequate wrist and pronator injection. Deep volar forearm adjacent to median nerve and radial artery; ultrasound guidance mandatory. Starting dose: 5–10 U IncoA.
	Lumbricals (palmar injection)	Finger adduction	5–15 U	15–45 U	Palmar surface injection. Addresses the finger adduction component of pill-rolling. Weakness at this dose range is negligible. Useful when thumb-to-index rolling persists despite proximal injection.
	Extensor digitorum communis (EDC)	Finger extension	Avoid routinely	—	FINGER DROP RISK. EDC weakness produces finger drop: inability to extend fingers at MCP joints, severely impairing hand opening, object release, and keyboard use. Do not include in routine templates. Use only for confirmed dystonic finger extension component on EMG, with explicit patient consent.

## Data Availability

No new data were created or analyzed in this study. Data sharing is not applicable.
